# Racehorse welfare across a training season

**DOI:** 10.3389/fvets.2023.1208744

**Published:** 2023-06-28

**Authors:** Rachel Annan, Leah E. Trigg, Jo Hockenhull, Kate Allen, Deborah Butler, Mathilde Valenchon, Siobhan Mullan

**Affiliations:** ^1^Bristol Veterinary School, University of Bristol, Bristol, United Kingdom; ^2^INRAE, CNRS, University of Tours, IFCE, UMR PRC, Nouzilly, France; ^3^UCD School of Veterinary Medicine, University College Dublin, Dublin, Ireland

**Keywords:** racehorse welfare, equine welfare, welfare assessment, equine welfare assessment, horse behavior, racehorse behavior, equine behavior, thoroughbred welfare

## Abstract

Racehorse welfare is gaining increasing public attention, however scientific evidence in this area is lacking. In order to develop a better understanding of racehorse welfare, it must be measured and monitored. This is the first study to assess racehorse welfare using scientific objective methods across a training season. The aim of this study was threefold, firstly to investigate welfare measures which could be used in the first welfare assessment protocol for racehorses. Secondly, to understand the effect that a racing and training season had on individual racehorses and thirdly to identify risk factors for both good and poor welfare. Thirteen racehorse training yards were visited at the beginning and the peak of the racing season in England. Behavioral observations along with individual environmental and animal-based welfare measures were carried out on 353 horses in 13 training yards selected for variability. In our sample the horses were generally in good physical health: 94% of horses recorded as an ideal body condition score, no horses had signs of hoof neglect and 77.7% had no nasal discharge. The overall prevalence of external Mouth Corner Lesions was 12.9% and was significantly higher for Flat racing than Jump racing horses. The majority of horses (67.5%) showed positive horse human interactions. When stabled 54.1% horses had physical social contact and nasal discharge was not associated with increased physical contact. The training season significantly affected Human Reactivity Tests, Horse Grimace Scale scores and time spent resting and feeding. A total of 14.5% of horses displayed stereotypic behavior on at least two occasions. Horses with windows in their stables spent more time surveying their surroundings. Overall, in this population of racehorses, horses spent around a third of their daytime feeding (33.7%) followed by time spent standing resting (22.6%). The welfare assessment protocol used in this study is suitable for use in industry to collect welfare data on racehorses.

## Introduction

Horse racing takes place in a variety of forms internationally. Racing integrity is governed by jurisdictions around the world and the concept of social license in equestrian sport and Thoroughbred (TB) horseracing continues to gain significant attention (1–3). Defined as the “level of approval or acceptance of society of a given activity” (4), social license relies on “sufficient trust and legitimacy” (5) from the public and society. One aspect of societal concern that jurisdictions are increasingly aiming to provide societal assurance on relates to equine welfare. It is therefore desirable to provide objective scientific evidence which can be used to monitor racehorse welfare, identify areas for improvements and education, increase transparency to address public concerns and highlight good practice (6).

The International Federation of Horseracing Authorities’ Minimum Horse Welfare Standards defines horse welfare as “the physical and mental state of a horse in relation to the conditions in which it lives and dies” (7). Welfare assessments are suggested to be most informative by both scientists (8, 9) and racing stakeholders alike (10), when they are multi-faceted, as no single measure will represent an animals’ overall welfare. The World Organization for Animal Health places importance on using animal-based measures primarily, with the addition of resource and management-based measures to give a relevant interpretation of the welfare status (11). Depending on the protocol, and the objective, animal welfare assessments can present stakeholders with baseline information ranging from individual animals, to data used as a population screening tool (12).

Equine welfare assessment has not progressed as quickly as other species and historically concentrated on working and neglected equines (13). In 2015, the Animal Welfare Indicators (AWIN) protocol for horses was developed (14) which considered leisure and sport horses for the first time. However, horses used for racing have a specific and intense exercise and management regime and tend to receive regular high level veterinary care (15), therefore existing welfare protocols were not entirely suitable for this population of horses.

While equine sports performance has received scientific attention the impact of a training season on a range of welfare indicators has not been studied. Typically, racehorses enter a training cycle where they spend a period of time in training, aiming to increase fitness via speed and/or endurance work, prior to entering a period where training is interspersed with racing, and then finally, following the most intensive training and racing period, the horses enter a period of complete rest or lower training load. Depending on the type of racing, horses enter their first training cycle at around 1.5–2 years old (Flat racing) or 3–4 years old (National Hunt or Jump racing). This cycle may be repeated seasonally or annually.

This study had three aims. Firstly, a range of input and outcome welfare indicators were trialed for feasibility in the first animal-based racehorse welfare assessment protocol suitable for use by racing jurisdictions aiming to better understand the welfare status of their population of horses. Secondly, we aimed to understand the effect of a training season on a range of welfare indicators of individual racehorses followed longitudinally at the beginning of the training season and the peak of the training/racing season. Finally, we aimed to be able to identify some of the risk factors for both poor and good welfare.

## Materials and methods

A racehorse-specific welfare assessment protocol was devised based on in depth stakeholder consultation (10, 16) and previously published equine welfare assessment methodologies (see Table 1). The welfare measures selected have been validated and are well developed and accepted indicators to assess equine welfare. The measures also needed to be acceptable to trainers, and the requirement that observations should be conducted by researchers without the need to handle the horses.

**Table 1:** Environmental and animal welfare measures.

Welfare measure	Recorded as	References
**Environmental measures**		
Type of yard	Flat or National Hunt Yard	
Window	Present or absent	
	*A window was defined as the horse being able to see out of the stable from an opening other than the stable door and could be either glass or open space.*	
Maximum amount of social contact	None, Visual, Sniff, Head, and Neck	
Social panel or grill	Present or absent.	
	*Social Panel or grill allowing horse to see and at least sniff horse in next stable.*	
Weaving bars	Present or absent	
	*On stable door*	
**Animal based measures**		
Body condition score (BCS)	0–5	(17)
	*Visually assessed from outside the stable for horses not wearing rugs.*	
Nasal discharge	Present (1 nostril or 2) or Absent	(18, 19)
	*May be watery or thick, transparent, yellow/green.*	
Ocular discharge	Present (1 eye or 2) or Absent	(18, 19)
External mouth corner lesions	0—Not Present	(20, 21)
	1—Present on one side	
	2—present on two sides	
	*Lesions, scaring, hard spots or hair loss.*	
Integument alterations on the head and neck	Present or absent	(22)
	*Alopecia, Skin lesion, Deep wound, Swelling.*	
	*Only lesions larger than a 1×2 cm^2^ area or more than 4 cm length (for linear lesions) are recorded.*	
Hoof neglect	Present—One or more hooves show one or more signs of neglect.	(22)
	Absent—None of the hooves shows any sign of neglect	
Horse Grimace scale	(0 = not present, 1 = moderately present, 2 = obviously present)	(23)
	*Each of the six facial action units of stiffly backwards ears, orbital tightening, tension above the eye area, prominent strained chewing muscles, mouth strained and pronounced chin, strained nostrils and flattening of the profile were scored using a 3-point scale*.	
Human reactivity tests	Positive, neutral, avoidance, negative, or ambiguous	(22)
Avoidance distance test (AD)		
Voluntary animal approach test (VAA)		

Thirteen racehorse training yards were visited for a two-day period at the beginning and again at the peak of the racing season resulting in a total of 4 days in each yard. Eight National Hunt (NH) yards were visited first in September 2018 and then in March 2019 and five Flat racing yards were visited first in January 2019 and finally in Summer 2019. Planned visits to an additional three flat yards had to be canceled due to an equine influenza outbreak in the United Kingdom. Yards were identified which represented a range in size, prize money won in the previous racing season and geographical location. Suitable racehorse trainers were invited to take part and inclusion in the study was voluntary. Each visit took place over two consecutive days, during which time observations were made of the environment and physical condition of horses in their stables on day one, and behavior of the horses on both days. As many horses per yard were observed as possible, but on larger yards was limited by the number that could be observed sequentially in a 15-min behavioral scan, given the layout of the yard. In those cases, the included horses were selected opportunistically but aiming to include a range in age and sex that was approximately representative of the yard. The visit at the peak of the season aimed to follow up on as many of the individual horses observed at the start of the season as possible and did not include new horses in the sample.

### Environmental observations

Environmental factors recorded were yard type, presence of a window, presence of weaving bars, presence of a social panel or grill between stables, maximum amount of social contact (see Table 1).

### Physical health observations

The physical animal-based measures (see Table 1) were assessed from outside the stable without handling the horse. Body Condition Score (BCS) was only recorded for horses that were not wearing rugs.

### Human–animal relationship tests

Two Human Reactivity Tests (HRT) were used to measure the human animal relationship: (1) the Avoidance Distance test (AD) and (2) the Voluntary Animal Approach test (VAA) (22). The AD test involved the observer approaching the stable door from 2.5 meters distance with an outstretched arm and noting the response behavior from the horse. The VAA test involves the assessor resting the hand on the stable door, as if to enter the stable, and recording the response behavior of the horse. Horses should be aware of the observer before starting the test. All horses were tested by one observer (RA). The HRT possible responses for both tests were modified for this study to include, Positive, Neutral, Avoidance, Negative and Ambiguous reactions from the horse, as opposed to “avoidance” or “no avoidance” and whether or not the horse approaches the door, respectively, for AD and VAA tests. Definitions of the possible responses to the test are categorized in Table 2.

**Table 2:** Behavior response categories for Human reactivity tests.

Response category	Positive	Neutral	Avoidance	Negative	Ambiguous
Behavior response shown	Ears pricked or move forward toward observer.	No change in behavior.	Horse moves body or head and neck away from observer.	Horse pins ears back.	Horse shows a mixture of positive and negative responses.
	Walked toward observer with ears pricked.			Horse moves toward observer with ears backwards.	
	Turned toward observer with ears pricked.			Horse shows teeth and moves head or body toward observer.	

### Horse Grimace scale

The Horse Grimace scale was conducted in-person by one observer (MV). Six facial regions were scored on a 0–2 scale according to the AWIN protocol. The sum of each facial regional score gave a total score of between 0 and 12 for each observation (22).

### Behavioral observations

Behavioral scan sampling was conducted on each horse in the sample, every 15 min from outside the stable door. The observation period covered most of the daily activity in each yard (from approximately 8 a.m. to 6 p.m. or one-hour post evening feed time), over two consecutive days, for each visit. Behaviors were recorded using a pre-determined and piloted ethogram by one of three trained observers (RA, MV, SM; see Table 3 for ethogram). Horses continued their usual routine that included exercising and other reasons for being out of the yard, and therefore during those times, observations were not able to be conducted. Definitions of stereotypic and abnormal behaviors are also described in Table 3. Where possible, when horses were turned out, close to the stable location, behavioral observations during turnout were conducted and recorded. When horses were not able to be observed while turned out they were recorded as absent.

**Table 3:** Ethogram for horse Behavior observation.

Behavioral activity	Description
Eat/drink	Horse is Masticating or swallowing forage or concentrate food. Or drinking water (24).
Resting in standing position	Standing on 3 or 4 legs, the eyelids and lips get droopy, eyes are at least partly closed (25).
Interested	Horse is interested in surroundings, watching people or other horses, listening to sounds, alert but calm.
Lying down	Horse is lying down in either sternal or recumbent position (26).
Stand	Standing with weight resting on all four legs. Not interested in surroundings but awake.
Elimination	Defecation or urination (27).
**Stereotypic behaviors**	
Crib biting and wind sucking	Horse grips onto a fixed object using incisor teeth, leans back onto hindquarters and contracts the strap muscle of the neck to bring the head into an arched position. Air is sometimes taken into the esophagus to produce a grunting sound (28) An oral stereotypic behavior.
Weaving	Lateral movement of the head, neck and shoulders from side to side in a rhythmic repetitive manner with alternation of the weight onto the contralateral foreleg with respect to the position of the head (28) A locomotor stereotypic behavior.
Box Walking	A locomotor stereotypic behavior, is the repetitive circular walking inside the stable (29).
**Abnormal behaviors**	
Wood chewing	Teeth are used to chew parts of the stable. Ingestion of wood may occur.
Lip smacking	Incisors are kept shut while lips are opened and closed.
Teeth scraping	Lips are curled back to expose incisors. Lateral and corner incisors are scraped back and forth against side of solid object.
Repetitive head movement	A repeated, relatively invariant sequence of movements with no obvious function adapted from Mason (30) including movements of the head such as headshaking, nodding and bobbing.
Repetitive oral	A repeated, relatively invariant sequence of movements with no obvious function using the teeth, lips or tongue (31).

Adapted from Young et al. (27).

### Data analysis

Descriptive analysis of both the environmental and animal-based welfare measures and the behavioral observations was carried out. The results report the proportion of horses in each category for individual measures and the mean proportion of observations spent exhibiting a behavior for behavioral observations. A horse was classified as displaying abnormal or stereotypic behavior, if it performed these behaviors on two or more occasions during data collection. Missing data were removed for analysis and all analyses only include horses who were present during both visits. Each of the welfare measures were modeled as response variables using the potential risk factors as explanatory variables to identify good or poor welfare in racehorses. Welfare measures were modeled using binomial Generalized Linear Mixed Models (GLMMs) and linear mixed-effects models for binomial and continuous measures, respectively. The models were fitted and analyzed using the lme4 package (32) in R (33). To account for non-independent and potentially clustered data as a result of repeated measures on the same horse and multiple horses at the same yard, individual horse ID and yard ID were modeled as random effects (34).

The response welfare indicator variables and potential explanatory risk factor variables can be seen in Table 4 along with the data type for statistical analysis. Response variables which were recorded in categorical or scale format were aggregated to binary variables for statistical analysis. From the Behavioral observation data, the mean proportion of observations in each Behavior category was treated as a response variable in the multivariable models.

**Table 4:** Response and potential risk factor variables as aggregated for statistical analysis.

Welfare measures		Notes
Ocular discharge	Binary	Present or not present
Nasal discharge	Binary	Present or not present
Mouth corner lesions	Binary	Present or not present
HRT approach	Binary	Positive or other
HRT stay/door	Binary	Positive or other
HGS score	Numerical	0–12
Behavior	Numerical	Proportion of observations showed behavior
**Potential risk factor explanatory variables**		
Visit	Binary	First or second visit
Type of yard	Categorical	NH or Flat training yard
Age	Numerical	Age in years when observed
Sex	Categorical	Male or female
Windows	Binary	Present or not present.
Maximum social contact	Categorical	Maximum amount of social contact—None, Visual, Sniff, Head, and Neck.
Social contact category	Categorical	Non-Physical = None or Visual
		Physical—Sniff or Head and Neck
Weaving bars	Binary	Present or not present on stable door
Bedding type	Categorical	Shavings or straw
Social panel or side grills	Binary	Side grills if present or not. Social Panel or grill allowing horse to see and sniff horse in next stable.

A backwards elimination process was used to select variables for each model. A Wald test provided the *p* values in the model summary output. *p* values of greater than 0.05 were used to guide the removal of explanatory variables for model comparison. Lowered Akaike information criterion (AIC) following the removal of variables showed an improved model fit and simplicity and determined the final model. Variance Inflation Factors were calculated using the Car package (35) to assess multicollinearity between explanatory variables. Models were also validated by visual inspection of the residuals and by using the DHARMa package (36) for residual diagnostics for mixed regression models (see Supplementary material).

In each of the final models, determined by AIC model selection, risk factors were considered significant if they had a *p*-value < 0.05 (37, 38). Odd ratios (OR), a measure of the association between an explanatory and response variable (39), or Estimated Marginal Mean (emmean), and Confidence Intervals (CI) were produced for each of the significant risk factor variables.

This study received University of Bristol ethical approval (VIN/18/033).

## Results

Data was collected for 353 horses (231 NH and 122 Flat) from eight NH and five Flat training yards in England, at both the beginning (visit 1) and at the peak (visit 2) of each racing season.

Initially 496 horses were assessed during visit 1 and 353 of these horses were also present during the second visit and were considered for the study. The 143 horses which had been assessed on the initial visit which were not present during the second visit were documented as absent along with the reason they were not present, given by a senior staff member or the trainer. Reasons for their absence included change of trainer or sold (*n* = 35, 7.1%), training break (*n* = 25, 5.1%), injury (*n* = 24, 4.8%), retirement (*n* = 14, 2.5%) and the reason for absence was unknown for 32 horses (6.5%). Thirteen horses (2.6%) had died. Only horses who were observed on both visits are included further in this analysis. The number of horses observed twice in each yard was as follows: 8 NH yards: 22, 36, 26, 38, 31, 23, 20, 35; 5 Flat yards: 33, 15, 34, 26, 14.

The median age of all horses was five (range 2–14) years old. At the peak of each racing season, the median age of Flat horses was two (range 2–9) years old and the median age of NH horses was six (range 2–14) years old. There were 209 geldings (177 NH, 32 Flat), 41 entire male horses (1 NH, 40 Flat) and 103 female horses (53 NH, 50 Flat).

### Environmental observations

The descriptive results from the environmental explanatory variables are shown in Table 5. All horses were individually stabled. Overall, 48.9% of observations were of horses in stables with windows, 54.1% with physical social contact, 57.2% without weaving bars and 42.9% with social panels or grills between stables. Most observations (85.7%) were of horses bedded on shavings as opposed to straw.

**Table 5:** Descriptive results of potential explanatory variables from both visits (Each horse was observed twice therefore there are two observations per individual horse).

	NH	Flat	Overall
	No. Horse observations (%)	No. horse observations (%)	No. horse observations (%)
**Sex**			
Total male	356 (77.1%)	144 (59.0%)	500 (70.8%)
Total female	106 (22.9%)	100(41.0%)	206 (29.2%)
**Windows**			
Window	224 (48.5%)	121 (49.6%)	345 (48.9%)
No window	238 (51.5%)	123(50.4%)	361 (51.1%)
**Social contact in stable**			
**Maximum social contact**			
None	0 (0.0%)	8 (3.3%)	8 (1.1%)
Visual	206 (45.6%)	110 (45.1%)	316(44.8%)
Sniff	212 (45.9%)	126 (51.6%)	338(47.9%)
Head and Neck	44 (9.5%)	0 (0.0%)	44 (6.2%)
**Social contact category**			
Non-physical contact	206 (44.6%)	118 (48.6%)	324 (45.9%)
Physical contact	256 (55.4%)	126 (51.4%)	382 (54.1%)
**Weaving bars**			
Present	105 (22.8%)	197 (80.7%)	302 (42.8%)
Not present	357 (77.3%)	47 (19.3%)	404 (57.2%)
**Side grills**			
Grill/social panel	185 (40.0%)	118 (48.4%)	303 (42.9%)
Solid walls	277 (60.0%)	126 (51.6%)	403 (57.1%)
**Bedding type**			
Shavings	361 (78.1%)	244 (100.0%)	605 (85.7%)
Straw	101 (21.9%)	0 (0.0%)	101 (14.3%)

### Physical health observations

The physical assessment results are shown in Table 6. Body condition score 3 (out of 5) was recorded for 94% of horses observed (range 2–3.5). No horses in this sample were observed with hoof neglect and in 94% of observations horses had no integument alterations greater than a 1 x 2cm^2^ area or more than 4 cm length (22) on their head or neck.

**Table 6:** Descriptive results of welfare response measures (each horse was observed twice therefore there are two observations per individual horse).

	NH number of horses (%)	Flat number of horses (%)	Range between yards %	Overall total number of horses (%)
**Nasal discharge**				
Present	91 (20.5%)	62 (25.5%)	7.9–50.0%	153 (22.3%)
Absent	352 (79.5%)	181 (74.5%)		533 (77.7%)
**Ocular discharge**				
Present	20 (5.1%)	12 (4.9%)	0–12.5%	32 (5.0%)
Absent	375 (94.9%)	231 (95.1%)		606 (95.0%)
**Mouth corner lesions**				
Present	36 (9.0%)	48 (20.1%)	0–29.6%	89 (12.9%)
Absent	366 (91.0%)	191 (79.9%)		603 (87.1%)
**Avoidance distance test (AD)**				
Positive	287 (65.4%)	171 (70.7%)	52.4–76.7%	458 (67.3%)
Neutral	105 (23.9%)	61 (25.2%)	14.7–35.7%	166 (24.4%)
Avoidance	15 (3.4%)	5 (2.1%)	0–6.0%	20 (2.9%)
Negative	24 (5.5%)	5 (2.1%)	0–18%	29 (4.3%)
Ambiguous	8 (1.8%)	0 (0.0%)	0–6.0%	8 (1.2%)
**Voluntary animal approach test (VAA)**				
Positive	279 (62.0%)	171 (70.7%)	51.9–73.3%	450 (65.0%)
Neutral	136 (30.2%)	65 (26.9%)	21.4–38.1%	201 (29.0%)
Avoidance	3 (0.7%)	1 (0.4%)	0–3.3%	4 (0.6%)
Negative	14 (3.1%)	5 (2.1%)	0–7.7%	19 (2.7%)
Ambiguous	18 (4.0%)	0 (0.0%)	0–13.5%	18 (2.6%)
**Horse Grimace scale score (0–12)**				
0	28 (7.7%)	6 (2.5%)	0–14.0%	34 (5.6%)
1	44 (12.0%)	31 (13.1%)	4.5–23.3%	75 (12.5%)
2	101 (27.6%)	62 (26.3%)	16.1–40.0%	163 (27.1%)
3	96 (26.2%)	82 (34.7%)	18.6–41.9%	178 (29.6%)
4	60 (16.4%)	42 (17.8%)	2.3–29.5%	102 (16.9%)
5	20 (5.5%)	5 (2.1%)	0–14.7%	25 (4.2%)
6	12 (3.3%)	4 (1.7%)	0–6.5%	16 (2.7%)
7	4 (1.1%)	4 (1.7%)	0–6.0%	8 (1.3%)
8	1 (0.3%)	0 (0.0%)	0–1.9%	1 (0.2%)

Overall, in 77.7% of observations horses showed no nasal discharge. The only variable which influenced nasal discharge was visit, with a greater number of horses with nasal discharge during visit 2 in the peak of the season compared to visit 1 at the start of the season (OR 0.433,95% CI 0.294–0.637; *P* ≤ 0.0001). There was no significant association between an increase in the amount of social contact and presence of nasal discharge (Physical Contact vs. Non-Physical Contact: OR 1.2, 95% CI 0.689–2.000, *p* = 0.49). Overall, in 95% of observations horses showed no ocular discharge and eye discharge was not influenced by any of the variables modeled. The overall prevalence of observations of external Mouth Corner Lesions (MCL) was 12.9% (NH 9.0%, Flat 20.1%). The odds of Flat horses having any MCL was 4.33 times the odds of NH horses (95% CI 1.9–9.88; *p* = 0.0005). The odds of female horses showing MCL was 0.41 times the odds of male horses (95% CI 0.176–0.967; *p* = 0.042). There was variation between trainers in the prevalence of MCL’s which ranged from 0% to 29.9%.

### Human reactivity tests

In the avoidance distance test the majority of horses showed a positive reaction (67.5%) and the odds of female horses showing a “positive” reaction (as opposed to neutral, negative, avoidance or ambiguous) was 0.52 times that of male horses (OR 0.52, 95% CI: 0.35–0.75, *p* ≤ 0.001). Significantly more horses showed a positive reaction when tested on visit 1 at the start of the season, compared to visit 2 at the peak of the season (OR 1.61, 95% CI: 1.15–2.25, *p* ≤ 0.005) and Flat horses were at greater odds of showing a positive reaction (OR 1.47, 95% CI: 1.01–2.14, *p* ≤ 0.04). In the voluntary animal approach test, 65% of observations recorded a positive response and the odds of Flat horses showing positive reactions were 1.5 times the odds of NH horses showing positive reactions (OR 1.5, 95% CI: 1.06–2.13, *p* = 0.024). Again, significantly more horses showed a positive reaction during visit 1 at the start of the season, than visit 2 at the peak (OR 1.84, 95% CI 1.33–2.55, *p* ≤ 0.001). There was no significant change in the prevalence of “neutral” or “avoidance/negative” reactions in either tests.

### Horse Grimace scale

**Figure 1:**
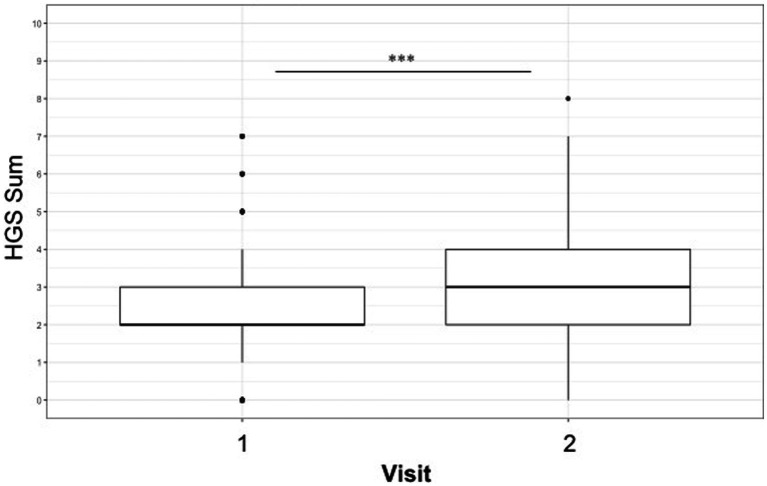
Boxplot showing increase in Horse Grimace scale score between early and peak season visits (emmean: Visit 1 vs. Visit 2, 2.38/3.22, ***p ≤ 0.001).

The mean HGS score across all observations was 2.70 (median 3) out of a maximum score of 12 (range 0–8). Significant model output results can be seen in Table 7. Significantly higher scores were recorded on observations conducted during visit 2 at the peak of the season compared with visit 1 at the start of the season (emmean; visit 1/visit 2; 2.38/3.22, *p* ≤ 0.0001) see Table 7 and Figure 1. Observations of female horses recorded significantly higher HGS compared to males (emmean; female/male; 3.00/2.64, *p* = 0.009) and HGS score increased with age.

**Table 7:** Linear mixed effect model output showing significant variables and includes estimated marginal mean (emmean) for Horse Grimace Scale scores.

Variable	emmean	CI	Estimate	*P*-value
Visit 1	2.38	2.11–2.65	−0.839	<0.0001
Visit 2	3.22	2.93–3.51		
Male	2.64	2.37–2.91	0.33	0.009
Female	3.00	2.66–3.27		
Age	2.80	2.53–3.07	0.06	0.04

### Behavioral observations

From the 353 horses which were observed on both visits at 13 yards, 43,730 observations were made over a total of 52 days. Overall, the most common behavior seen was eat/drink behavior (33.7%) followed by standing resting (22.6%). The proportion of observations of NH and Flat horses as well as the range between yards, and individual horses can be seen in Table 8.

**Table 8:** Percentage of observations in each behavior category.

Behavior	Percentage of observations in each behavior category				
	NH horses %	Flat horses %	Overall %	Range between yards %	Range between individual horses %
Abnormal or stereotypic	3.6	2.1	3.1	0.6–5.7	0–48
Absent	13.8	5.5	10.9	3.1–21.8	0–90
Eating or drinking	31.2	39.3	33.7	20.5–46.9	0–70
Interested	10.4	8.3	9.6	4.0–13.5	0–43
Lie down	1.0	3.96	1.9	0.0–5.6	0–25
Resting in standing position	23.8	20.5	22.6	11.8–32.4	0–56
Social interaction	0.6	0.6	0.6	0.0–2.0	0–10
Turned out	5.8	0.1	3.8	0.0–43.2	0–75
With or restrained by human	3.9	4.4	4.1	0.9–14 0.7	0–36

The proportion of observations that horses were recorded eating or drinking ranged from 0% to 70% between horses. The significant model outputs can be seen in Table 9. Significantly fewer observations were made of horses eating or drinking during visit 2, at the peak of the season (emmean; visit 1/visit 2: 0.35/0.32, *p* ≤ 0.0001) and when they had a window (emmean; window/no window; 0.33/0.35, *p* = 0.015).

**Table 9:** Results from logistic regression models showing significant variables influencing racehorse behavior.

Explanatory variable	emmean (proportion of observations)	OR and 95% confidence interval	*P*-value
**Eat and drink behavior**			
Visit 1/visit 2	0.35/0.32	1.19 (1.14–1.24)	<0.0001
No window/window	0.35/0.33	1.09 (1.02–1.17)	0.0150
**Resting behavior**			
Visit 1/visit 2	0.20/0.25	0.78 (0.74–0.81)	<0.0001
**Interested**			
No window/window	0.08/0.10	0.75 (0.67–0.84)	<0.0001
Physical contact/no physical contact	0.09/0.08	0.81 (0.71–0.92)	0.0008
Visit 1/visit 2	0.094/0.087	1.08 (1.01–1.15)	0.0175
**Lying down**			
Type—Flat/NH	0.02/0.01	3 (1.8–5.02)	<0.0001
Age (estimate)	0.0102	Estimate = − 0.25	<0.0001
Physical contact/no physical contact	0.01/0.008	1.46 (1.14–1.88)	0.003
**Abnormal or stereotypic**			
No weaving bars/weaving bars	0.022/0.014	1.57 (1.24–1.98)	0.0002

National Hunt and Flat horses were recorded to be standing resting for 24 and 21% of observations, respectively. Only visit significantly influenced resting behavior, with more frequent observations during visit 2 at the peak of the season (emmean; visit 1/visit 2 = 0.20/0.25; *p* ≤ 0.0001).

The overall proportion of observations when horses were recorded as “Interested” was 9.6%. Horses who did not have a window in their stable were significantly less likely to be observed as interested than those who did have a window (emmean; No Window/Window = 0.08/0.10; *p* ≤ 0.001). A significantly higher proportion of observations of horses interested was recorded during visit 1 at the start of the season compared to visit 2 at the peak (emmean: visit 1/visit 2 = 0.094/0.087; *p* = 0.018). There were significantly fewer observations of horses being recorded as interested when they had physical contact (emmean; Physical Contact/No Physical Contact = 0.08/0.10, *p* ≤ 0.001).

Horses were observed lying down for 1.9% of observations, and there was variation between yards with frequency of observations ranging from 0% to 5.6%. Significantly more observations were of horses lying down when they were Flat horses (emmean: Flat/NH = 0.02/0.01, *p* ≤ 0.0001), younger horses (estimate = −0.2445, *p* ≤ 0.0001), and had physical social contact (emmean: Physical Contact/No Physical Contact = 0.01/0.008, *p* = 0.003).

### Abnormal and stereotypic behavior

Out of 353 horses observed, 51 horses performed a stereotypic behavior (as previously defined as crib biting, windsucking, weaving or box walking) at least twice during one of the two visits, giving a prevalence of stereotypic behavior of 14.5% of racehorses observed. Of those horses who performed stereotypic behaviors at least twice, the mean proportion of observations where the horse performed stereotypic behaviors was 13% (range 1% to 48%). A further 72 horses displayed abnormal behavior on at least two occasions and in total 123 horses (34.8%) displayed any abnormal or stereotypic behavior. Horses with weaving bars were observed performing abnormal or stereotypic behaviors significantly less often than those without weaving bars (emmean; No weaving bars/weaving bars = 0.022/0.014, *p* ≤ 0.001).

### Turn-out

Turnout practices varied considerably between yards and the mean percentage of scans where horses were turned-out during our observations ranged from 0% to 43%, see Table 8. In some yards, horses were turned out for a number of hours after being ridden or overnight, while in others there was no turnout available. In 3.8% of all scan sample behavior observations, horses were turned out. Horses in NH yards (5.8%) were turned out during more observations than horses in Flat yards (0.1%). For individual horses, the number of observations spent turned out ranged from 0% to 75%. Horses were turned out in groups, pairs and individually in fields, paddocks and small individual enclosures.

## Discussion

This is the first study to conduct comprehensive assessments covering a range of environmental, physical and behavioral welfare measures on the same racehorses at the start and peak of a training and racing season. Our results suggest that a racing season indeed represents a form of challenge for a racehorse’s welfare state, but that some specific factors—such as opportunities for social contacts and increased visual horizons—have potential to help horses overcome the challenge. In addition, our study demonstrated the feasibility and use of a set of welfare measures which provided an insight into the welfare of high physical performance racehorses over time.

### Impact of a racing season on racehorse welfare

A number of horses (28%) were not available during the second visit for a variety of reasons reported in the results, including, change of yard, holiday, injured and 13 horses had died. It is possible that these horses struggled with the increased intensity of the training season.

At the peak of the season, positive reactions to a human approach decreased (human reactivity test), HGS scores increased, horses spent less time feeding but more time resting, and nasal discharge increased (but with almost no severe forms observed) compared to the beginning of the season. Racehorses are trained intensively in order to perform to their optimum athletic potential with the ultimate goal to win races. The training and exercise program differs between trainers and the type of racing they will compete in (Flat or NH), but will involve an increasingly intensive conditioning program of the cardiovascular and musculoskeletal systems during the training season (40). On average horses will run 4.51 (Flat 5.08, NH 3.31) (41) times during the racing season. Horses will normally have a break at the end of each season, when they spend a number of weeks at grass and are not exercised, before starting the training cycle again. As the exercise and training intensity increased over the racing season, horses need to rest for longer, as our results confirmed. The decrease in feeding behavior is consistent with studies which have shown a decrease in appetite of racehorses as fitness increases (42). Loss of appetite is a welfare concern within the racing industry as it could be linked to health issues such as gastric ulceration. One way to prevent this is to provide free access to grass and/or forage (e.g., hay). In the present study, a majority of horses were provided with *ad libitum* access to grass/forage which is a positive practice regarding equine welfare (43).

During both of the Human Reactivity tests, which were carried out by the same observer (RA) on both visits, horses were at greater of odds of showing a positive reaction at the start of the season compared to the peak of the season, with no significant change in the prevalence of “neutral” or “avoidance/negative” reactions. The horse human relationship is important both for health and safety and for the animal’s welfare state (44). The horse’s reaction will be dependent on its past experiences with humans, its own temperament as well as the approach and skills of the human involved. The daily interactions of horses and their caregivers has been shown to influence how the horse perceives humans in general (44), therefore importance must be placed on the regular attitude and demeanor of racehorse grooms in order to develop positive horse human relationships. Management systems typically vary between training yards with some trainers choosing to maintain the same groom and or rider and horse relationship, while others will not, which is often dependent on staff supply levels (16). This further highlights the need to promote a horse centered attitude across all staff in order to promote positive welfare (45). The most common reaction to both of the Human Reactivity Tests, across the season, was positive (67.3% and 65% for AD and VAA respectively), followed by neutral reactions (24.4% and 29.0%) and a smaller number of horses showed an avoidance, negative or ambiguous reaction. Results from this study were similar to a validation study for these tests which found the majority of horses responded positively but that horses kept in sub optimal welfare conditions showed more avoidance, defensive or aggressive behaviors when approached (46, 47). However, researchers found no significant differences when repeating the tests at 3-month intervals whereas in this racehorse population, horses showed more positive reactions in the early part of the season. As the prevalence of neutral reactions did not significantly change accordingly over the training season, we cannot exclude that this decrease in positive reactions might indicate early signs of welfare alteration for some individual horses. It highlights the importance of noticing any change of attitude to humans to monitor welfare. However, as the prevalence of negative reactions did not significantly increase neither and remain generally at a low level in our study, the main reasons for the decrease in positive reactions here could be therefore that horses became accustomed to people in the yard as the season progressed and were less interested in an unknown human, or that as exercise increased, horses spent more time resting, which was the case. Flat horses reacted more positively than NH horses in both tests. Flat horses begin racing at an earlier age compared to NH horses and tend to have a shorter racing career (48). More positive reactions from Flat horses might be explained by the fact they have spent less time in training and are therefore more interested in an approaching human.

Horse Grimace Scale (HGS) scores increased from the beginning to the peak of the season and with age, suggesting an influence from the increased intensity of training during the season and over the horse’s career impacting the HGS score. Scores were also higher for female horses. The HGS has been used to indicate levels of pain in horses with laminitis and dental pain and undergoing castration surgery (23, 49–51). The mean HGS score in our study was 2.7 (out of a possible 12; median = 3). This is the first study that has used the HGS to assess racehorses in training and these horses have scored higher than a population of leisure and sport horses assessed using the AWIN welfare protocol where only 2% of horses scored ≥ 2 (52). In other studies investigating the HGS, control horses, who were described as not in pain, were scored ≤ 2 (23, 49) and horses scored > 5 in studies where painful procedures such as acute laminitis and castration were assessed (23, 50). It is not clear what small differences in HGS scores at the lower end of the scale mean for the horses themselves. It could be that some racehorses may be experiencing low level chronic pain possibly due to their rigorous training programs, and the HGS could be used to identify these horses before more serious injuries appear, however more research in this area is needed.

### Risk factors for racehorse welfare

Our results highlight the importance of the opportunities for social contact for racehorse welfare. All horses were individually housed in a variety of types of stables with differing amounts of social contact both between, and within training yards. Thereby, 54.1% of horses had physical social contact when stabled, which meant they could at least sniff another horse through a social panel or grill between stables, a low wall or at the stable door. This level of social contact is higher than that reported in the leisure horse population where 39%–44% had physical contact while stabled (52, 53). In our study, access to physical contact (sniff or head and neck) was associated with more lying down, suggesting a greater level of relaxation and quality of sleep. Indeed, horses are more likely to lie down in a sternal or recumbent position when they feel safe and there are social companions nearby (54). Furthermore, horses can also only enter paradoxical Rapid Eye Movement (REM) sleep when they lie down in a recumbent position (55), making lying down an essential activity. Providing opportunities for social contacts appeared therefore positive for the welfare of the present racehorse population.

As a social species (56), the importance of well-established bonds with known conspecifics has been well documented as an essential welfare need for horses (57–59). Our results suggest that efforts to increase social contacts for racehorses have been made within the racing industry and has positive repercussions. However, improvement is still required as the majority of social contacts we observed were still restricted to nose-to-nose contacts, usually through bars. Previous research indeed showed that even if horses are intrinsically motivated to access any level of social contacts (60, 61), full-body contacts are necessary to the establishment of social relationships (62, 63). As we observed in our study, racehorses are still mainly housed in individual stables during training, as protection from injury and cross-contamination of pathogens is a major concern. Yet, in 2020, the International Federation of Horseracing Authorities published minimum horse welfare standards (7), which included “*opportunities to bond with other animals as a desirable condition to optimise horse welfare*.” The Irish Thoroughbred Welfare Council (64) also included social contact as an important aspect of horse welfare in their recently published welfare principles. Finally, social contact was highlighted as “*the best life*” scenario for racehorses by stakeholders within the racing industry (10). Altogether, these elements suggest that pursuing the efforts to increase social contacts for racehorses would be an effective and concrete way to improve equine welfare in the industry.

The presence of nasal discharge is a common equine welfare measure (65) and can be a sign of a respiratory condition which may limit equine athletic performance and is therefore a concern for trainers. Indeed, avoiding disease and managing good health, was one of the primary welfare challenges identified by racehorse trainers and veterinary surgeons when asked to discuss racehorse welfare (16). Respiratory conditions have been reported as an important reason racehorses lost days of training, second only to lameness (66, 67). In this study 78% of horses showed no nasal discharge and the majority of discharge was transparent when observed. Anecdotal evidence has shown that racehorse trainers attempt to limit the spread of infections between horses by reducing physical social contact, which could be seen as a welfare concern (68). Results from this study showing no significant association between increased social contact and the presence of nasal discharge should encourage racehorse trainers to safely increase social contact between horses.

Another factor that appears to influence racehorse daily behavior is the presence of windows in the stable. Overall, in this population of racehorses, horses spent around a third of their daytime feeding (eating and drinking, 33.7%) followed by standing resting (22.6%). The time budget of free and semi-free ranging horses has been reported between 29.8–66.5% of time spent foraging and 8.1–36.6% resting, with domesticated stabled horses spending between 10% and 64% eating or foraging and 15.6%–68% resting (69–71). The activity budget of our racehorses falls within these ranges. The wide range of time budgets can be explained by the variation of habitat and management routines in each population. Racehorses in this study spent 9.6% of observations “Interested,” with NH horses spending more time interested than their Flat counterparts. Interested behavior was defined as being alert but calm in their surroundings and horses who had a window in their stable were observed interested more often than those who did not have a window. These results support studies which found that windows and increasing the visual horizon for the horse, provided environmental enrichment for stabled horses and improved welfare (72–74). Horses with windows in their stables were often observed looking out of them surveying the activity going on around the yard. Therefore, windows should be a consideration, if horses must be stabled, to increase positive welfare.

Horses were seen lying down an average of 1.9% of total observations and, as previously mentioned, horses who had physical social contact were at higher odds of lying down than those who had non-physical social contact (visual or none), suggesting that these horses felt more relaxed lying down in their stables. Flat and younger horses were also seen lying down more often. Sleep in a recumbent position has been documented as being especially important to younger horses and necessary to develop memory recall (71, 75, 76). In humans, good quality sleep is vital for advanced athletic performance and recovery (77) and although research in sleep and equine performance is lacking it could be considered to have a similar effect. It would therefore be beneficial for racehorse trainers to utilize stabling which encourages quality sleep behavior to improve equine welfare and potentially performance. For example horses have been shown to lie down in a recumbent position for longer periods when they are stabled in larger loose boxes (78) and bedded on straw (79) as opposed to shavings.

### Abnormal behavior and stereotypies

The prevalence of abnormal and stereotypic behaviors in our study suggest there is still a need for improvement for racehorse welfare, as for other equine populations. The prevalence of stereotypic behavior (crib biting, wind sucking, weaving, and box walking) (80–82) of racehorses in this study was 14.5%. Previous studies investigating stereotypical behavior in horses in general found prevalences of between 6.2%–32.5% (82, 83) and 6%–15% for Thoroughbred horses specifically (80, 84). Stereotypies have been universally linked with poor animal welfare (31), however, as they are generally developed early in life, the presence of stereotypies cannot be used as a measure of the current welfare status of an individual, or necessarily reflect the management practices experienced at that time (85). The presence of stereotypical behavior could however be used as a measure of the welfare of a population of animals over time. A range of causes of stereotypic behavior have been reported including stress, boredom, neurological triggers, the inability to express essential behaviors and genetics (31, 81, 86–88). Evidence would suggest separate causes for oral (crib biting and wind sucking) and locomotor (box walking and weaving) stereotypies (89) and a breed propensity for stereotypic behavior in Thoroughbred horses has been suggested (84). Other behaviors which are considered abnormal in the horse include wood chewing, tongue movements, lip smacking and others described in Table 3. The amount of social contact, having grills between stables, age, type of horse (Flat vs. NH) and sex did not influence abnormal or stereotypic behavior. Horses without weaving bars were observed performing stereotypic or abnormal behaviors significantly more often, than those with weaving bars. Weaving Bars (WB) were present on the stable doors of 42.8% of horses, with the majority of NH horses not having WB and the majority of Flat horses having WB present. Weaving bars are used to prevent the horse performing the weaving behavior, over the stable door, however previous evidence has shown them to be unsuccessful as horses will continue to weave inside the stable (29). To sum up, the prevalence of abnormal and stereotypic behaviors in the present study is in accordance with other findings and confirm stereotypies as an important issue with animals kept under the care of humans. Monitoring such behaviors is a prime interest at the population-level. Different leads have been mentioned in the discussion to prevent their appearance and expression during training, including offering more social and sensory stimulations to the racehorses. However, a specific focus on the early-life challenges encountered by racehorses (e.g., early social environment, weaning, career start) would also contribute to reducing abnormal and stereotypic behaviors at the population-level. Individual coping styles of horses should also be considered. Horses performing explicit or abnormal behaviors may have a proactive coping style (90), however horses which do not show overt behaviors may too have experienced poor welfare and display a passive response (58) which caregivers should be aware of.

### Other welfare indicators

A set of basic physical indicators have been tested in our study and most of them showed a lack of inter-yard and inter-horse variability since the vast majority of horses were observed with good scores. For instance, body condition scores and signs of hoof neglect revealed no problem, which was expected for high performance horses. However, thin racehorses and those with poor hoof condition are occasionally reported to the BHA as welfare concerns. It is therefore useful for jurisdictions to have data to know whether these are isolated cases or more widespread when seeking to provide societal assurance on reported welfare issues. The low prevalence in our study of some physical welfare issues highlights the need to have additional types of welfare parameters for such equine populations, as we investigated here, to assess racehorse welfare (e.g., behavioral parameters). However, one exception might be the external Mouth Corner Lesions (MCL) at the lip commissures. The overall prevalence was 12.9% in this study and was significantly higher in Flat horses compared to NH horses (20.1% vs. 9.0), and there was a variation between trainers in the prevalence of MCL’s ranging from 0.0% to 29.6%. A study of Danish competition horses reported 9.2% visible oral lesions or blood across a range of equestrian disciplines, examined using a similar inspection method to the current study. Recent studies in event horses found 52% of horses examined had acute oral lesions in the bit area after the cross country event (91), and 83% and 90% of Standardbreds and Finnhorses, respectively, of Finnish trotting horses had bit-related lesions (92), however these studies also included internal mouth corner lesions and an internal mouth examination which the current study did not. Female horses were significantly associated with less mouth corner lesions which differs from previous studies (92). The variability between trainers in the occurrence of MCL’s might be explained by rider skill level or approaches to riding across training yards or possibly the various types of tack and bits used. The type of bit used has been significantly associated with the presence of MCL in a number of studies (20, 91–93). MCL might therefore constitute an interesting physical indicator to monitor in the future within the industry.

## Conclusion

The welfare assessment protocol used in this study proved to be suitable for use in industry to collect welfare data on racehorses in a training yard environment. The protocol uses non-invasive measures which can be carried out on a sample of horses in a relatively short period of time. Assessors should have equine experience and be trained in welfare assessment but not necessarily be veterinary professionals. The protocol identified good physical health and frequent positive horse human reactions and was sensitive enough to detect changes in welfare in the population over the course of the training season. In addition, it was sufficiently comprehensive to determine some risk factors for poor and good welfare. Areas have also been highlighted for future welfare improvements, industry education and industry-wide monitoring, although it should be noted the yards in this study were selected for variability and do not necessarily reflect the whole racehorse industry in the United Kingdom or beyond.

In a subsequent phase, most of the measures have been successfully trialed by non-scientist assessors from the industry in more than 70 yards, showing their wider applicability. However, further welfare measures which impact racehorse welfare such as racecourse injuries and lameness were not included in this protocol. Reasons for these omissions included that the focus of the study was on the time horses spent in training yards as opposed to the racecourse and the need for measures that were acceptable to the industry at that time. When assessing racehorse welfare, using a holistic assessment protocol which encompasses both environmental and animal-based indicators ensures a full picture of welfare can be gained. If racehorses are expected to work at the upper limit of equine athletic ability, it is important that, overall, they experience many positive experiences in order to ensure a positive welfare balance.

## Data availability statement

The datasets presented in this article are not readily available because the datasets analyzed during this study are not publicly available. Requests to access the datasets should be directed to rachel.annan@bristol.ac.uk.

## Ethics statement

The animal study was reviewed and approved by University of Bristol Ethics Committee.

## Author contributions

RA, MV, and SM were involved in the study design and planning. RA, MV, DB, and SM were involved in data collection. RA and LT were involved in data analysis and interpretation. JH and KA provided advice on the manuscript. RA wrote the paper with all authors contributing to critical revisions. All authors contributed to the article and approved the submitted version.

## Funding

This project was funded by The Racing Foundation - Grant Number 183/212.

## Conflict of interest

The authors declare that the research was conducted in the absence of any commercial or financial relationships that could be construed as a potential conflict of interest.

## Publisher’s note

All claims expressed in this article are solely those of the authors and do not necessarily represent those of their affiliated organizations, or those of the publisher, the editors and the reviewers. Any product that may be evaluated in this article, or claim that may be made by its manufacturer, is not guaranteed or endorsed by the publisher.
