# Supplementary material for: Racehorse welfare across a training season
Source: Front Vet Sci. 2023 Jun 28;10:1208744. doi: 10.3389/fvets.2023.1208744 (PMC10336241; doi:10.3389/fvets.2023.1208744)
Supplement: Supplementary file 1 [file Data_Sheet_1.docx]

Supplementary Material

Racehorse welfare across a training season

Rachel Annan^1*^, Leah E Trigg^1^, Jo Hockenhull^1^, Kate Allen^1^, Deborah Butler^1^, Mathilde Valenchon^1, 2,✝^, Siobhan Mullan^1,3,✝^

*** Correspondence:** [Rachel.Annan@bristol.ac.uk](mailto:Rachel.Annan@bristol.ac.uk)

# Supplementary Figures and Tables

The following information includes summary results from each of the final selected models for each response variable with significant explanatory variables. Diagnostic residual plots from the DHARMa package are also presented for each model. The QQ plot residuals did not detect significant issues with residuals in the majority of models with the exception of the Horse Grimace Scale, Resting and lying down behavior, which detected small deviations from normality, but no major issues were observed. Some deviations from uniformity have been detected in the diagnostics for the Lying Down model, most likely due to the small number of horses who were observed lying down in the sample population.

## Mouth Corner Lesions Model

Supplementary Table 1. Summary results of the final selected model of Mouth Corner lesions in racehorses (estimates, standard error, t values and p values).

|  | Estimate | Std. Error | z value | Pr(>\|z\|) |
| --- | --- | --- | --- | --- |
| (Intercept) | -2.8367 | 0.6507 | -4.36 | 1.30E-05 |
| TypeNH | -1.4648 | 0.4212 | -3.478 | 0.000505 |
| SexCatMale | 0.8873 | 0.4364 | 2.033 | 0.042026 |

**Supplementary Figure 1.** Diagnostics from the DHARMa package and graphical summary of results for the final model of Mouth Corner Lesions.

## Horse Grimace Scale Model

Supplementary Table 2. Summary results of the final selected model of Horse Grimace Scale in racehorses (estimates, standard error, t values and p values).

|  | Estimate | Std. Error | z value | Pr(>\|z\|) |
| --- | --- | --- | --- | --- |
| (Intercept) | 1.4479 | 0.23918 | 103.87858 | 2.28E-08 |
| Age | 0.06094 | 0.0308 | 147.69099 | 0.04976 |
| SexCatMale | -0.32536 | 0.12405 | 320.32467 | 0.00914 |
| Visit | 0.83863 | 0.09945 | 275.8698 | 1.91E-15 |

Supplementary Figure 2. Diagnostics from the DHARMa package and graphical summary of results for the final model of Horse Grimace Scale.

## Human Reactivity Test – Avoidance Distance (AD) Test Model

**Supplementary Table 3.** Summary results of the final selected model of Human Reactivity Test – Avoidance Distance (AD) Test. (estimates, standard error, t values and p values).

|  | Estimate | Std. Error | z value | Pr(>\|z\|) |
| --- | --- | --- | --- | --- |
| (Intercept) | 0.7961 | 0.2075 | 3.837 | 0.000125 |
| TypeNH | -0.386 | 0.1909 | -2.021 | 0.043234 |
| SexCatMale | 0.6644 | 0.1945 | 3.416 | 0.000636 |
| Visit2 | -0.4764 | 0.1716 | -2.776 | 0.005504 |

Supplementary Figure 3. Diagnostics from the DHARMa package and graphical summary of results for the final model of Human Reactivity Test – Avoidance Distance (AD) Test.

## Human Reactivity Test – Voluntary Animal Approach (VAA) Test Model

Supplementary Table 4. Summary results of the final selected model of Human Reactivity Test – Voluntary Animal Approach (VAA) Test. (estimates, standard error, t values and p values).

|  | Estimate | Std. Error | z value | Pr(>\|z\|) |
| --- | --- | --- | --- | --- |
| (Intercept) | 1.2214 | 0.1822 | 6.704 | 2.03E-11 |
| TypeNH | -0.4057 | 0.1791 | -2.265 | 0.023513 |
| Visit2 | -0.6079 | 0.1665 | -3.652 | 0.000261 |

**Supplementary Figure 4.** Diagnostics from the DHARMa package and graphical summary of results for the final model of Human Reactivity Test – Voluntary Animal Approach (VAA) Test.

## Eat/Drink Behavior Model

Supplementary Table 5. Summary results of the final selected model of Eat/Drink Behavior in racehorses (estimates, standard error, t values and p values).

|  | Estimate | Std. Error | z value | Pr(>\|z\|) |
| --- | --- | --- | --- | --- |
| (Intercept) | -0.56256 | 0.08911 | -6.313 | 2.73E-10 |
| Visit2 | -0.15454 | 0.02087 | -7.404 | 1.32E-13 |
| Window_Bin1 | -0.08534 | 0.03512 | -2.43 | 0.0151 |

Supplementary Figure 5. Diagnostics from the DHARMa package and graphical summary of results for the final model of Eat/Drink Behavior.

## Rest Behavior Model

Supplementary Table 6. Summary results of the final selected model of Rest Behavior in racehorses (estimates, standard error, t values and p values).

|  | Estimate | Std. Error | z value | Pr(>\|z\|) |
| --- | --- | --- | --- | --- |
| (Intercept) | -1.51553 | 0.12874 | -11.772 | <2e-16 |
| Visit2 | 0.2637 | 0.02268 | 11.625 | <2e-16 |
| Age | 0.02656 | 0.01387 | 1.916 | 0.0554 |

Supplementary Figure 6. Diagnostics from the DHARMa package and graphical summary of results for the final model of Rest Behavior.

## Interested Behavior Model

Supplementary Table 7. Summary results of the final selected model of Interested Behavior in racehorses (estimates, standard error, t values and p values).

|  | Estimate | Std. Error | z value | Pr(>\|z\|) |
| --- | --- | --- | --- | --- |
| (Intercept) | -2.51872 | 0.10334 | -24.374 | 2.00E-16 |
| Visit2 | -0.07614 | 0.03205 | -2.376 | 0.017522 |
| Window_Bin1 | 0.28353 | 0.05777 | 4.908 | 9.20E-07 |
| PhysicalContactVisualNone | 0.21305 | 0.06337 | 3.362 | 0.000773 |

Supplementary Figure 7. Diagnostics from the DHARMa package and graphical summary of results for the final model of Interested Behavior.

## Lying Down Behavior Model

Supplementary Table 8. Summary results of the final selected model of Lying Down Behavior in racehorses (estimates, standard error, t values and p values).

|  | Estimate | Std. Error | z value | Pr(>\|z\|) |
| --- | --- | --- | --- | --- |
| (Intercept) | -2.19048 | 0.19402 | -11.29 | 2.00E-16 |
| Age | -0.44256 | 0.03681 | -12.023 | 2.00E-16 |
| PhysicalContactVisualNone | -0.36534 | 0.1315 | -2.778 | 0.00546 |

Supplementary Figure 8. Diagnostics from the DHARMa package and graphical summary of results for the final model of Lying Down Behavior.

## Abnormal Behavior Model

Supplementary Table 9. Summary results of the final selected model of Abnormal Behavior in racehorses (estimates, standard error, t values and p values).

|  | Estimate | Std. Error | z value | Pr(>\|z\|) |
| --- | --- | --- | --- | --- |
| (Intercept) | -3.77972 | 0.08878 | -42.575 | 2.00E-16 |
| WeavingBars_Bin1 | -0.4485 | 0.12011 | -3.734 | 0.000188 |

Supplementary Figure 9. Diagnostics from the DHARMa package and graphical summary of results for the final model of Lying Down Behavior.
